# HIV counseling, testing, and test result receipt among East African women of reproductive age using recent national health surveys

**DOI:** 10.3389/frph.2024.1305671

**Published:** 2024-02-07

**Authors:** Bewuketu Terefe

**Affiliations:** Department of Community Health Nursing, School of Nursing, College of Medicine and Health Sciences, University of Gondar, Gondar, Ethiopia

**Keywords:** counseling, East Africa, HIV testing, receipt result, women

## Abstract

**Introduction:**

One of the most important public health concerns is still the Human Immunodeficiency Virus (HIV) and acquired immunodeficiency syndrome (AIDS), particularly in developing countries. Although HIV testing is an important step in both prevention and treatment, its uptake remains low, and no study has looked into the scale of HIV counseling, testing, and test result receipt among East African women all at the same time. Therefore, this study aimed to investigate HIV counseling, testing, and test result receipt, as well as the factors that influence them, among East African women.

**Methods:**

This analysis used Demographic and Health Survey data collected from 10 East African countries between 2011 and 2022. We examined the coverage of HIV counseling, testing, and test result receipt among East African women, as well as other characteristics. To select candidate factors and identify significant explanatory variables related to the outcome variable, binary and multiple logistic regression analyses were conducted, and the results were presented using adjusted odds ratios (AORs) with 95% confidence intervals. In the binary and multiple logistic regression analyses, *P* values of less than or equal to 0.2 and <0.05 were used to assess significant variables, respectively.

**Results:**

A total of 41,627 weighted women included to this study. HIV counseling, testing, and test result receipt among East African women were found to be 77.86% (95% CI = 77.46, 78.26). Being 25–34 years old (AOR = 1.13, 95% CI, 1.06, 1.21), 35–49 years old (AOR = 1.15, 95% CI, 1.05, 1.26) as compared to 15–24 years old women, primary education (AOR = 1.75, 95% CI, 1.64, 1.86), secondary/higher education level (AOR = 1.96, 95% CI, 1.82, 2.13) as compared to not educated women, poor, (AOR = 1.22, 95% CI, 1.14, 1.29), middle wealth (AOR = 1.12, 95% CI, 1.04, 1.21) as compared to rich wealth index, mass media exposure (AOR = 1.29, 95% CI, 1.22, 1.35), 3–5 parity (AOR = 1.29, 95% CI, 1.21, 1.37), more than 5 parity (AOR = 1.46, 95% CI, 1.33, 1.61) as compared to <3 parity, health institution delivery (AOR = 1.65, 95% CI, 1.53, 1.76), were associated positively with the outcome variable respectively. However, being married (AOR = 0.79, 95% CI, 0.72, 0.87), not using contraceptive (AOR = 0.58, 95% CI, 0.51, 0.61), and traditional contraceptive method user (AOR = 0.47, 95% CI, 0.41, 0.54) as compared to modern users were associated negatively with outcome variable respectively.

**Conclusion:**

This study found that HIV counseling, testing, and test result receipt are still unsatisfactory. Strengthening maternal health services such as institutional delivery, family planning, and women's empowerment, as well as changing mass media and taking advantage of these opportunities, will boost the region's coverage of HIV counseling, testing, and obtaining results.

## Introduction

HIV/AIDS is a major global public health concern, with Sub-Saharan Africa having the highest prevalence of infection (1, 2). According to the USAIDS, 2023 estimate, around 39 million individuals were newly infected with HIV in 2022 and lived with the virus (3). Similarly, AIDS-related illnesses claimed the lives of almost 630,000 people this year (3). Of them, two thirds originated in Africa, the continent where most deaths occur (4). However, just 36.9 million persons globally were HIV positive in 2017, with Eastern and Southern Africa making over half of that number (19.6 million) (2, 5). Reports state that while new infections in children decreased by 52% from 310,000 in 2010 to 150,000 in 2019, new infections worldwide decreased by about 40% from 2.9 million in 1997 to 1.7 million in 2019. However, Sub-Saharan Africa (SSA) accounted for over 61% of new infections among children, most likely as a result of mother-to-child HIV transmission (MTCT) (6, 7). The aforementioned various year reports demonstrate that the fight against HIV/AIDS is still ongoing worldwide, particularly in developing nations.

In many countries, it is now national policy for all pregnant women to get provider-initiated testing and counseling during antenatal care (ANC). This is known as “opt-out” HIV testing. In “opt-out” testing, HIV testing, like hemoglobin tests, is included in the standard ANC care package for all pregnant women. All ANC patients are offered the test and counseled on the advantages and disadvantages of knowing their HIV status during pregnancy. However, testing is still voluntary, and women have the option to decline if they so desire. Women are more likely to accept HIV testing if their health care physician counsels and encourages the practice (5, 8). Because of the effects of HIV/AIDS, the United Nations 2020 Global AIDS Update announced significant progress in the fight against the HIV epidemic (6–8). Since the UNAIDS 90-90-90 effort, which aims to provide HIV testing and treatment to the great majority of HIV-infected persons by the end of 2020, more than 80% of people living with HIV were aware of their status, and approximately 67% were receiving antiretroviral therapy (9).

Eastern Africa is the world's second-most HIV/AIDS-affected region, behind Southern Africa. Over the past 20 years, the region's overall frequency has decreased—Kenya saw a reduction in prevalence from 14% to 5% but new worries about infection among the most vulnerable individuals are starting to emerge (10). A number of studies have determined that the following variables are responsible for the higher rate of HIV transmission: types of residencies; exposure to mass media; exposure to HIV/AIDS education; having taken an HIV test; lack of awareness of HIV; poor knowledge; education level; wealth index; inability to access ART prophylaxis; poor adherence to ART; absence of clinic-based HIV education and counseling (11–16).

Additionally, gender raises the risk of HIV infection. In 2015, 25% of new HIV infections among adults were among young women, but in SSA, 56% of new infections were among women (17). The WHO and the United Nations Program on HIV/AIDS released guidelines for provider-initiated HIV testing and counseling in 2007 in response to the low testing rates (18). In order to facilitate enhanced availability of HIV prevention, treatment, and care, the World Health Organization (WHO) recommended in 2015 that quick diagnostic HIV testing be carried out by trained lay practitioners (19). The risk variables associated with HIV testing have not been thoroughly studied, and there exist regional and gender-specific variations.

Although community-owned interventions are critical for increasing HIV testing and counseling for women, the country's little research in this area focuses solely on individual drivers, leaving the combination effect of counseling, testing, and receiving the results. Previous research focused on either on counseling, testing, or just getting the results. Therefore, utilizing recent demographic and health surveys conducted in East Africa, the goal of this study was to assess the prevalence, and factors related to HIV counseling, testing, and obtaining the results among women in reproductive age in that region.

## Methods

### Study setting, and period

The data used in this study comes from the most recent standard Demographic and Health Survey (DHS) dataset for East African states. The dataset covers 10 years from 2012 to 2022 and includes data from ten East African countries: Burundi, Ethiopia, Kenya, Comoros, Madagascar, Malawi, Mozambique, Rwanda, Tanzania, Uganda, Zambia, and Zimbabwe. The study utilized a standard dataset to acquire a substantial sample size that is representative of the population source and all circumstances (20). On a global basis, DHS collects analogous statistics. The surveys feature large sample sizes, are population-based, and are nationwide in scope (20). Eastern Africa comprises 14 countries across the Horn of Africa, the Indian Ocean islands, and the Great Lakes region. These countries share similar economic, social, and environmental challenges. Furthermore, they are worried about their ability to achieve the targets set by the Millennium Development Goals (21). East Africa is the region of the African continent located in the horn and east of the Sahara Desert. They are home to 486,766,759 people and cover an area of 6,667,493 Km2 (2,574,332 square miles), accounting for 6.03% of the global population.

### Data source and study population

We utilized DHS data from 2012 to 2022, specifically from individual record (IR) files. Out of the 14 eastern countries where DHS surveys were conducted during this period, only approximately 10 were included in our study due to the absence of information concerning the outcome variable in the remaining surveys. After incorporating the data from each country, we had a total weighted sample of 41,627 pregnant women and an unweighted sample of 41,881 reproductive-age pregnant women who had undergone HIV counseling and testing and received their results. These samples were used for the final analysis (Table 1).

**Table 1:** Countries, sample size, and survey year of demographic and health surveys included in the analysis for 10 East African countries.

Country	Survey year	Sample size (weighted)	Frequency (weighted)
Burundi	2016/2017	6,505	15.63
Ethiopia	2016	1,855	4.46
Comoros	2012	282	0.68
Madagascar	2021	1,055	2.54
Malawi	2016/2017	8,309	19.96
Mozambique	2011	4,389	10.54
Rwanda	2019/2020	4,634	11.13
Uganda	2016	6,815	16.37
Zambia	2018	4,869	11.70
Zimbabwe	2015	29,142	7.00

### Sample size determination and sampling method

Recent Demographic and Health Survey (DHS) reports were available for approximately 12 out of the 13 nations in East Africa. Each of the surveys conducted in the listed nations used the most up-to-date conventional census framework. In the DHS samples, the areas are usually divided into urban and rural regions within each administrative geographic area. During the first round of sampling, enumeration areas (EAs) were selected based on their size within each stratum using a probability-proportional approach. In the second step of sampling, a systematic sampling method was used to select a specific number of homes in the designated EAs. After the household listing, systematic sampling with equal probability was employed to choose a certain number of households from within the defined cluster (20).

### Data management and statistical analysis

We used STATA version 17 to extract, clean, and recode the variables in our study. To account for the differential probability of selection in the DHS data sampling procedure, we weighted the data using sample weights during any statistical analysis. This ensured that the survey results were representative. For the bivariable analysis, we considered variables with a p-value of 0.2 for the multivariable analysis. In the multivariable logistic model, we provided the Adjusted Odds Ratio (AOR) with a 95% confidence interval to determine the associated factors of HIV counseling, testing, and obtaining findings. For categorical data, descriptive studies such as frequency count and proportion were utilized to summarize the descriptive data. Bivariable logistic regression was used to determine candidate variables for multiple logistic regression. A logistic model was fitted using the variance inflation factor to investigate multicollinearity among the independent variables. To assess the overall fitness of the final regression model, the Hosmer and Lemeshow tests were used. The statistical significance of the final model was set at *p* 0.05. We tested it for the assumption of multilevel model analysis using the intra-class correlation (ICC) coefficient because the data could be hierarchical, but it was 3%, which did not meet the minimal threshold to perform it. As a result, it was determined that traditional logistic regression was preferred.

### Variables of the study

#### The outcome variable

The outcome variable of this study was the number of pregnant women who received counseling on HIV, tested for an HIV test during antenatal care, and received the results of the test. The outcome variable was then recategorized as Yes = “1” if the women were counseled on HIV, tested for HIV, and received the test results accordingly, and No = “0” if the women did not receive either of them. This classification and analysis were made according to the guide in the DHS statistics book (20).

#### The independent variables

Independent variables: Various maternal-related factors were included. These included maternal age, educational status, types of places of residence, marital status, household wealth index, current employment status, mass media exposure, place of delivery, number of health visits, total children born, under-five children, contraceptive utilization, distance to the health facility, knowledge of HIV/AIDS, sex of the household head, and breastfeeding status.

## Results

### Sociodemographic characteristics of the study participant

In this study, a total of 41,627 pregnant women of reproductive age were included in East African countries. About 18,761 (45.07%) of the study women were between 25 and 34 years of reproductive age. Regarding marital status, more than half of the women (27,177, or 65.29%) were married. Concerning place of residence types, 32,156 (77.25%) had rural areas as their place of residence, educational status 22,140 (53.18%) had primary education, wealth index 8,752 (21.03%) belonged to the poorest households, and breastfeeding status, 28,374 (68.17%) of mothers were currently breastfeeding. Furthermore, about 36,958 (88.78%), 22,200 (53.33%), and 26,343 (63.28%) women who have given birth at health institutions did not have mass media exposure (either listening to the radio, watching television, or reading magazines or newspapers) and are currently employed, respectively. However, slightly more than half of the 21,694 (52.12%) and the majority of the 24,994 (60.05%) mothers did not utilize any method of contraception, and they reported that the distance to the health facility is a major problem in accessing it. About 16,185 (38.88%) of women have given birth to approximately 3–5 children; of these, 35,065 (84.23%) of mothers have under-five children. However, 39,511 (94.92%) of mothers did not have comprehensive knowledge of HIV/AIDS, and almost 32,345 (77.70%) of mothers visited health facilities once a year. Similarly, about 16,629 (39.95%) of females have reported that the distance from the health facility was a major problem in accessing services (Table 2).

**Table 2:** Socio-demographic and maternal related characteristics of respondent's HIV counselling testing and received results among reproductive age women in east African countries (weighted *n* = 41,627): based on east African the recent DHS data.

Variables	Categories	Frequency	Percentage
Age in years	15–24	14,666	35.23
	25–34	18,761	45.07
	35–49	8,200	19.70
Residence	Urban	9,472	22.75
	Rural	32,156	77.25
Mothers’ educational status	No education	7,496	18.01
	Primary	22,140	53.18
	Secondary and higher	11,990	28.81
Mother is employed	No	15,284	36.72
	Yes	26,343	63.28
Wealth index	Poorest	8,752	21.03
	Poorer	8,450	20.30
	Middle	7,948	19.09
	Richer	8,230	19.77
	Richest	8,247	19.81
Mass media exposure	No	22,200	53.33
	Yes	19,427	46.67
Under five children number	No	1,271	3.06
	1–2	35,065	84.23
	More than two	5,291	12.71
Total children ever born	<=2	18,458	44.34
	3–5	16,185	38.88
	>=5	6,984	16.78
Contraceptive methods	No method	21,694	52.12
	Traditional methods	1,071	2.58
	Modern methods	18,860	45.31
Number of health visits in the past 12 months	Once	32,345	77.70
	More than one	9,282	22.30
Distance from health facility	Not a big problem	24,994	60.05
	A big problem	16,629	39.95
Currently breastfeeding	No	13,249	31.83
	Yes	28,374	68.17
Place of delivery	Home	4,669	11.22
	Health facility	36,958	88.78
Marital status	Never married	2,751	6.61
	Married	27,177	65.29
	Divorced/widowed	11,694	28.10
Comprehensive HIV knowledge	No	39,511	94.92
	Yes	2,116	5.08
Sex of the household head	Male	31,612	75.94
	Female	10,015	24.06

### Factors associated with HIV counseling, testing and receiving the results among reproductive age women in East Africa

The odds of HIV counseling, testing, and receiving the results increased by 13% (AOR = 1.13, 95% CI: 1.06–1.21) and 15% (AOR = 1.15, 95% CI: 1.05–1.26), respectively, among women aged 25–34 years and 35–49 years, compared to women aged 15–24 years. Additionally, compared to uneducated women, those who had completed primary and secondary/higher education showed (AOR = 1.75; 95% CI: 1.64–1.86) and (AOR = 1.96; 95% CI: 1.82–2.13) higher chances of receiving HIV counseling, testing, and results. Similarly, about the household wealth index, mothers from poor and middle households had a higher likelihood of receiving counseling, testing, and results compared to women from wealthy households, with odds of (AOR = 1.22, 95% CI, 1.14–1.29) and (AOR = 1.12, 95% CI, 1.04–1.21) respectively. 29% of mothers with mass media exposure received proper HIV counseling, testing, and results, with odds of (AOR = 1.29, 95% CI = 1.22, 1.35), compared to their counterparts. Mothers with 3–5 children and more than five children had a higher likelihood of receiving counseling, testing, and results, with odds of (AOR = 1.29, 95% CI: 1.21–1.37) and (AOR = 1.46, 95% CI: 1.33–1.61) respectively, compared to mothers with fewer than three children. Married mothers had a 21% lower likelihood of not receiving HIV counseling, testing, and results (AOR = 0.79, 95% CI: 0.72–0.87) compared to never-married women. On the other hand, mothers who gave birth at health institutions had a higher likelihood of receiving HIV counseling, testing, and results compared to those who gave birth at home. Compared to users of modern contraceptive methods, those who used no method and traditional contraceptive methods had a lower tendency to receive HIV counseling, testing, and results, with odds of (AOR = 0.58, 95% CI, 0.51–0.61) and (AOR = 0.47, 95% CI, 0.41–0.54) respectively (Table 3).

**Table 3:** Multiple logistic regression analysis results on determinants of HIV counselling testing and received results among reproductive age women in east Africa, based on the recent DHS data (weighted *n* = 41,627).

HIV counseling and testing and received results	No, *n* (%)	Yes, *n* (%)	AOR	Std. errs.	*z*	*P* > z	95% CI
Variables							
Maternal age							
15–24	3,542 (24.15)	11,122 (75.85)	1				
25–34	3,963 (21.12)	14,800 (78.88)	1.13	0.037	3.63	0.0001	**1.06**, **1.21**
35–49	1,709 (20.84)	6,491 (79.16)	1.15	0.055	2.93	0.003	**1.05**, **1.26**
Maternal education							
Not educated	2,378 (31.73)	5,118 (68.27)	1				** **
Primary	4,592 (20.74)	17,547 (79.26)	1.75	0.055	17.54	0.0001	**1.64**, **1.86**
Secondary & higher	2,244 (18.71)	9,747 (81.29)	1.96	0.079	16.66	0.0001	**1.82**, **2.13**
Employment status							
No	3,531 (23.10)	11,754 (76.90)	1				** **
Yes	5,683 (21.58)	20,658 (78.42)	1.05	0.026	2.00	0.046	**1.01**, **1.11**
Wealth index							
Poor	3,951 (22.97)	13,252 (77.03)	1.22	0.039	5.98	0.0001	**1.14**, **1.29**
Middle	1,814 (22.83)	6,133 (77.17)	1.12	0.042	2.99	0.003	**1.04**, **1.21**
Rich	3,449 (20.93)	13,028 (79.07)	1				
Residence							
Urban	1,800 (19.00)	7,672 (81.00)	1				
Rural	7,414 (23.06)	24,740 (76.94)	1.05	0.035	1.60	0.110	0.99, 1.13
Mass media exposure							
No	5,545 (24.98)	16,657 (75.02)	1				
Yes	3,669 (18.89)	15,756 (81.11)	1.29	0.034	9.50	0.0001	**1.22**, **1.35**
Total children born							
<3	4,350 (23.57)	14,107 (76.43)	1				** **
3–5	3,336 (20.61)	12,849 (79.39)	1.29	0.043	7.56	0.0001	**1.21**, **1.37**
>5	1,528 (21.88)	5,456 (78.12)	1.46	0.072	7.70	0.0001	**1.33**, **1.61**
Marital status							
Never married	623 (22.62)	2,130 (77.38)	1				** **
Married	6,134 (22.57)	21,045 (77.43)	0.79	0.039	−4.79	0.0001	**0.72**, **0.87**
Divorced/widowed	2,457 (21.01)	9,238 (78.99)	0.93	0.049	−1.45	0.148	0.84, 1.03
Place delivery							
Home	1,512 (32.38)	3,157 (67.62)	1				
Health facility			1.65	0.059	13.98	0.0001	**1.53**, **1.76**
Number of health visits							
Once	7,093 (21.93)	25,252 (78.07)	1				
More than one	2,122 (22.86)	7,161 (77.14)	0.95	0.027	−1.67	0.095	0.91, 1.01
Contraceptive methods							
No method	5,770 (26.60)	15,924 (73.40)	0.58	0.015	−21.14	0.0001	**0.55**, **0.61**
Traditional	295 (27.48)	778 (72.52)	0.47	0.034	−10.54	0.0001	**0.41**, **0.54**
Modern	3,149 (16.70)	15,711 (83.30)	1				
_cons			1.39	0.106	4.41	0.0001	1.21, 1.62

Bold variables significant at *p* value <0.05 in the multiple regression.

## Discussion

This study aimed to determine the prevalence and associated factors of HIV counseling, testing, and test result receipt among East African women of reproductive age. The final model included age, educational level, wealth index, media exposure, use of contraception, access to healthcare facilities, marital status, parity, and work status as factors connected to HIV counseling, testing, and receipt results in East Africa. HIV counseling and testing are widely recognized as essential components of all HIV preventive strategies. Testing can identify individuals who are HIV positive and in need of treatment and care, as well as those who are HIV negative and should be referred for HIV prevention and care. Regular HIV testing will be crucial for pre-exposure prophylaxis-based preventive strategies. HIV counseling and testing not only help women, couples, and families learn their HIV status and receive personalized risk reduction counseling and further care based on their status, but they can also help communities overcome the stigma and discrimination associated with HIV/AIDS (22, 23). This study employs a nationally representative cross-sectional sample of women to evaluate the factors that influence HIV counseling, testing, and receiving results among East African women of reproductive age. As a result, the findings of this study suggest that various variables are associated with women of reproductive age.

The odds of HIV counseling, testing, and receiving the results were increased by 13% more among women whose ages are 25–34 years old and 35–49 years old, respectively, as compared to women whose ages are younger. This survey's findings are comparable with those of other research, demonstrating that HIV counseling, testing, and receiving outcomes improve with age (24–27). Previous research on HIV/AIDS awareness and knowledge among women of reproductive age has discovered that as women get older, their chances of being aware of and knowledgeable about HIV/AIDS also increase. This, in turn, may result in a higher likelihood of them seeking HIV counseling and testing and receiving their test results as outlined in the protocol (28). This is most likely due to the older age group's lower fear of stigma and prejudice from society about HIV counseling and testing uptake compared to the younger age group (29).

Furthermore, when comparing educated women to those without formal education, it was determined that those who had completed primary and secondary/higher education had a 1.75% and 1.96% higher likelihood of receiving HIV counseling, testing, and obtaining the results. This discovery supports previous research that suggests women with higher levels of education are more likely to achieve positive outcomes (1, 25, 26, 30, 31). This result highlights the importance of education and higher wealth in promoting HIV testing and counseling. This may be because educated women and women from wealthier households tend to have greater HIV awareness and knowledge (28). Formal education also encourages healthcare-seeking behavior, which can contribute to a higher quality of life (32, 33).

Similarly, regarding household wealth index, mothers who came from poor and middle households have shown a higher likelihood of being counseled and tested and receiving the results as compared to women who came from rich households. This finding agrees with several studies done elsewhere (24–26, 34). Due to significant distances from health facilities and challenging transportation networks in Africa, impoverished households' mothers may find it more difficult to get HIV counseling, testing, and receiving findings than wealthier and more educated women (35). Also, women with greater incomes and educational levels are more likely to seek maternal health care services, have women's autonomy, and are closer to information (36, 37).

Mothers who have been exposed to mass media have a higher rate of properly receiving HIV counseling and testing results (29%) compared to those who have not been exposed. Mass media is an effective and affordable tool for disseminating health information and promoting health activities to a wide audience (38). Previous research has demonstrated that the media can positively influence HIV testing rates (39–41), as well as other critical public health issues (42). Turk and colleagues' 2017 impact data analysis found that the media can positively influence HIV test rates as well as unhelpful/stigmatizing attitudes and behaviors (38).

Mothers who have given from 3 to 5 and more than five children have revealed a higher likelihood of being counseled, tested, and receiving the result accordingly as compared to mothers who have less than three children, respectively. Consistent with earlier studies in SS (43). The number of children a woman had was shown to be related to HIV testing, with women who had more children having a higher likelihood of testing for HIV than those who did not have children. This finding can be linked to women's access to healthcare during pregnancy, delivery, and postpartum (43–45).

Married mothers have shown a 21% lower likelihood of not having HIV counseling, testing, or receiving the results when compared to never-married women. Previous research found that divorced and separated people were more likely to be tested for HIV than married people (45–47). Divorced and separated people were more than four times more likely than married people to die from HIV/AIDS. Single or never-married people were 13 times more likely than married people to die from HIV/AIDS (48, 49). Because of their larger sexual network, single/never married and divorced/separated people are at a higher risk of contracting HIV/AIDS and dying from with (50). Marriage appears to impose a sort of social control (49, 51), which helps to limit a spouse's number of sexual partners. Individuals at high risk (single and divorced) should be targeted with behavior change communications to encourage them to seek HIV testing, which is the cornerstone of HIV prevention (52).

On the other hand, when it comes to the place of delivery, mothers who have given birth at healthcare institutions have shown a higher likelihood of receiving HIV counseling, being tested, and obtaining results compared to mothers who gave birth at home. In comparison to users of modern contraceptive methods, those who do not use any method or opt for traditional contraceptive methods have shown a lower inclination towards HIV counseling, testing, and receiving the results accordingly. This is because mothers who have utilized maternal reproductive health services such as delivering at a healthcare facility and using modern contraception are more likely to receive information from healthcare professionals about HIV/AIDS, STIs, and available health treatments. As a result of these and other factors, they may enhance their health-seeking behavior, attitudes, and prevention methods (43, 53, 54).

### Strength and limitation of the study

The utilization of nationally representative surveys from different East African nations to evaluate HIV counseling, testing, and getting results coverage and its related factors is a fundamental strength of this study. As a result, we believe our findings are applicable to other SSA nations. Another significant strength of this study is the integration of all three (HIV counseling, testing, and obtaining results). The study data was gathered using conventional and verified data gathering procedures and could be affected by the number of countries. Nonetheless, the following limitations apply to the study. So, first of all, a cause-and-effect relationship cannot be proved due to the cross-sectional nature of the study. Second, the DHS may be biased by memory because it depends on self-reported statistics. Issues including accessibility to treatment, factors pertaining to health professionals, other cultural, biological or clinical related variables, and support programs were not included in the analysis. Finally, we had to rely on surveys conducted at different locations within the chosen countries due to data constraints and availability.

## Conclusion

This study found that HIV counseling, testing, and test result receipt among East African women was low. The study also indicated that the status of HIV counseling, testing, and test result receipt was determined by several factors. Generally, more than three-fourths of women of reproductive age had been counseled, tested, and had their HIV examination. After controlling for confounders, determinants such as age, educational attainment, wealth index, mass media exposure, contraceptive utilization, health facility delivery, marital status, parity, and employment status were associated with HIV counseling, testing, and receiving results in East Africa. As a result, in order to reduce the spread of HIV/AIDS, efforts must be made to address the obstacles that impede HIV counseling, testing, and obtaining results among women. Any stakeholder involved in HIV/AIDS prevention and control in any country should take into account factors such as educational levels and wealth status. These factors significantly influence the uptake of HIV counseling, testing, and obtaining results in East Africa. To improve the utilization of maternal health services, it is crucial to support economically marginalized women and enhance HIV/AIDS education in working and educational environments. Additionally, strengthening maternal health services, including institutional delivery, family planning, and women's empowerment, will be beneficial. This can be achieved by changing mass media and making use of the opportunities available to increase the coverage of HIV counseling, testing, and obtaining results in the region. Ultimately, these efforts will help improve women's health and reduce the incidence of HIV/AIDS in East Africa.

## Data Availability

The raw data supporting the conclusions of this article will be made available by the authors, without undue reservation.
